# Metformin stimulates IGFBP-2 gene expression through PPARalpha in diabetic states

**DOI:** 10.1038/srep23665

**Published:** 2016-03-24

**Authors:** Hye Suk Kang, Ho-Chan Cho, Jae-Ho Lee, Goo Taeg Oh, Seung-Hoi Koo, Byung-Hyun Park, In-Kyu Lee, Hueng-Sik Choi, Dae-Kyu Song, Seung-Soon Im

**Affiliations:** 1 Department of Physiology, Keimyung University School of Medicine, Daegu 42601, Korea; 2 Department of Internal Medicine, Keimyung University School of Medicine, Daegu 41931, Republic of Korea; 3 Department of Life Sciences, Ewha Womans University, Seoul 03760, Republic of Korea; 4 Division of Life Sciences, Korea University, 145 Anam-Ro, Seongbuk-Gu, Seoul, 02841, Republic of Korea; 5 Department of Biochemistry, Chonbuk National University Medical School, Jeonju, Jeonbuk 54896, Republic of Korea; 6 Division of Endocrinology and Metabolism, Department of Internal Medicine, Kyungpook National University School of Medicine, Daegu, 41944, Republic of Korea; 7 National Creative Research Initiatives Center for Nuclear Receptor Signals, Hormone Research Center, School of Biological Sciences and Technology, Chonnam National University, Gwangju, 61186, Republic of Korea

## Abstract

The anti-diabetic drug, metformin, exerts its action through AMP-activated protein kinase (AMPK), and Sirtuin (*Sirt1*) signaling. Insulin-like growth factor (IGF)-binding protein 2 (IGFBP-2) prevents IGF-1 binding to its receptors, thereby contributing to modulate insulin sensitivity. In this study, we demonstrate that metformin upregulates *Igfbp-2* expression through the AMPK-Sirt1-PPARα cascade pathway. In the liver of high fat diet, *ob/ob*, and *db/db* mice, *Igfbp-2* expression was significantly decreased compared to the expression levels in the wild-type mice (*p* < 0.05). Upregulation of *Igfbp-2* expression by metformin administration was disrupted by gene silencing of *Ampk* and *Sirt1*, and this phenomenon was not observed in *Pparα*-null mice. Notably, activation of IGF-1 receptor (IGF-1R)-dependent signaling by IGF-1 was inhibited by metformin. Finally, when compared to untreated type 2 diabetes patients, the metformin-treated diabetic patients showed increased IGFBP-2 levels with diminished serum IGF-1 levels. Taken together, these findings indicate that IGFBP-2 might be a new target of metformin action in diabetes and the metformin-AMPK-Sirt1-PPARα-IGFBP-2 network may provide a novel pathway that could be applied to ameliorate metabolic syndromes by controlling IGF-1 bioavailability.

In biological fluids, the insulin-like growth factor binding protein (IGFBP) family proteins can complex with both IGF-I and II, and are regulators of IGF actions on metabolism and growth^1^,^2^. Currently, there are six mammalian IGFBPs designated IGFBP-1–6 have been characterized^3^ and newly IGFBP-7 was identified as a member of the IGFBP superfamily^4^. The primary function of the IGFBPs is to restrict the bioavailability of IGF-1 in target tissues^5^. Among the IGFBPs, IGFBP-2 modulates IGF-1 bioactivity by interacting with IGF-1^6^. IGFBP-2 is highly expressed in the liver, adipocytes, and central nervous system, and is involved in metabolic homeostasis, insulin resistance, diabetes, and obesity^7^,^8^,^9^. Moreover, IGFBP-2 is suggested to be used as a marker protein for metabolic dysfunction^10^,^11^. IGFBP-2 is the abundant in blood, and has been shown to play a role in preventing insulin resistance and diet-associated obesity in mice^9^. However, the regulatory mechanism of IGFBP-2 expression and its clinical relevance to diabetic states in mice and humans remain poorly understood.

Sirtuin 1 (Sirt1), an NAD^+^-dependent protein deacetylase, is involved in controlling glucose, lipid homeostasis, aging, inflammation, and circadian regulation of metabolism and cellular processes^12^. It regulates metabolic homeostasis by deacetylating crucial transcriptional factors such as peroxisome proliferator-activated receptor α (PPARα), PPARγ, farnesoid X receptor (FXR), liver X receptor α (LXRα), PPARγ coactivator 1-α (PGC-1α), and p53^13^.

PPARα belongs to the nuclear receptor superfamily; it functions as a transcription factor and plays crucial roles in the regulation of varieties of metabolic dysfunctions related to inflammatory response, glucose and lipid metabolism^14^. PPARα is expressed in various tissues including the liver, adipose tissue, heart, kidney, and intestine^14^,^15^. PPARα is activated during fasting or by ligands; PPARα induces fatty acid oxidation and gluconeogenesis^16^. In addition, PPARα is known to suppress inflammation and to preserve insulin sensitivity^17^. It also interacts with the retinoid X receptor, and the resulting heterodimer promotes the transcriptional activation of target genes by binding to the consensus PPAR response element (PPRE) on target gene promoters^18^. Further, PPARα regulates cancer metabolism by attenuating IGF-1R signaling and Akt phosphorylation in various cancer cells^19^,^20^. However, a potential link between the Sirt1-PPARα axis and IGF-1 signaling system has not been elucidated yet.

The antidiabetic drug, metformin, is widely used for the treatment of type 2 diabetes. Metformin attenuates hepatic glucose production and triglycerides accumulation by ameliorating hyperglycemia and fatty oxidation in the liver^21^,^22^,^23^. Metformin also improves hepatic dysfunction by stimulating liver kinase B-1, which promotes the expression of AMP-activated protein kinase (AMPK) in the liver^24^. Because AMPK functions as a potential intracellular energy sensor and a master regulator of metabolic homeostasis, understanding the mechanisms of its activation by various physiological stimuli or therapeutic drugs and several hormones including adiponectin and leptin^25^ are of utmost importance in developing anti-diabetic drugs.

In this study, we elucidated the potential role of IGFBP-2 in diet-induced obesity or diabetic mice. Our results demonstrated that metformin controls *Igfbp-2* gene transcription through the AMPK-Sirt1-PPARα signaling pathway. Moreover, we showed that regulation of the Sirt1-PPARα-IGFBP-2 signaling cascade by AMPK activator represents a novel pathway that could be applied to ameliorate metabolic syndromes by controlling IGF-1 homeostasis.

## Results

### Metformin increases Igfbp-2 gene expression in primary hepatocytes

**Figure 1:**
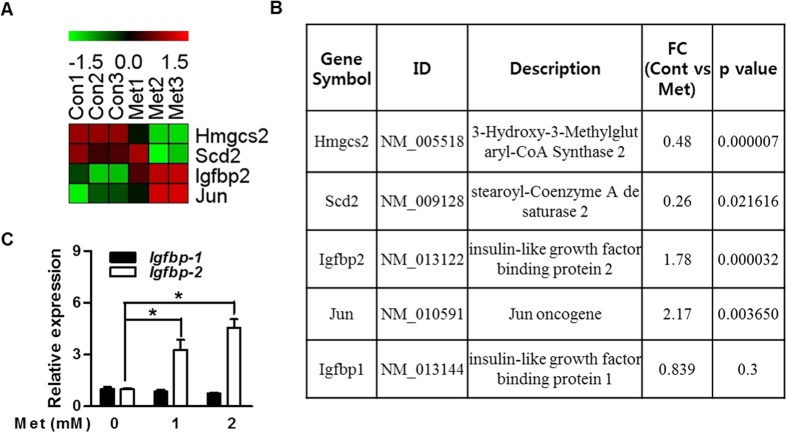
Metformin increases IGFBP-2 gene expression in primary hepatocytes. DNA microarray analysis of gene expression in the metformin-treated primary hepatocytes. The ratios of gene profiles were presented by either heatmap (**A**) or gene expression pattern (**B**). (**B**) Representative values from the DNA microarray. Relative *Igfbp-1* and *Igfbp-*2 mRNA levels with metformin treatment compared to control group in primary hepatocytes. (**C**) *Igfbp-2* expression in primary hepatocytes stimulated by metformin (12 h) for the indicated dose. *p < 0.05, **p < 0.01 vs. untreated control.

*Igfbp-2* gene expression is decreased in diabetic condition and increased when insulin resistance was improved in diabetic animal model^10^. We first performed DNA microarray analysis and compared global mRNA expression patterns in the primary hepatocytes by gene profiling to find putative target genes of metformin. Among genes that exhibited significant change in expression of 1.5-fold or higher, IGFBPs gene with control genes are listed in Fig. 1A. Because metformin as an anti-diabetic drug is able to improve insulin sensitivity in diabetic patients^26^, we have speculated that metformin might be responsible for the regulation of *Igfbp-2* gene expression and biological function. The expression level of *Igfbp-2* was significantly increased in the metformin-treated primary hepatocytes (Fig. 1B). Next, to confirm microarray data under *in vitro* conditions, we performed quantitative polymerase chain reaction (qPCR) analysis on metformin-exposed mouse primary hepatocytes. As shown in Fig. 1C, *Igfbp-2* expression was markedly increased by metformin compared to untreated control (*p* < 0.05); however, *Igfbp-1* expression was not induced by metformin treatment (Fig. 1C). Together, these findings demonstrate that metformin upregulates *Igfbp-2* expression *in vitro*.

### Hepatic Igfbp-2 gene expression in HFD, ob/ob, and db/db mice

**Figure 2:**
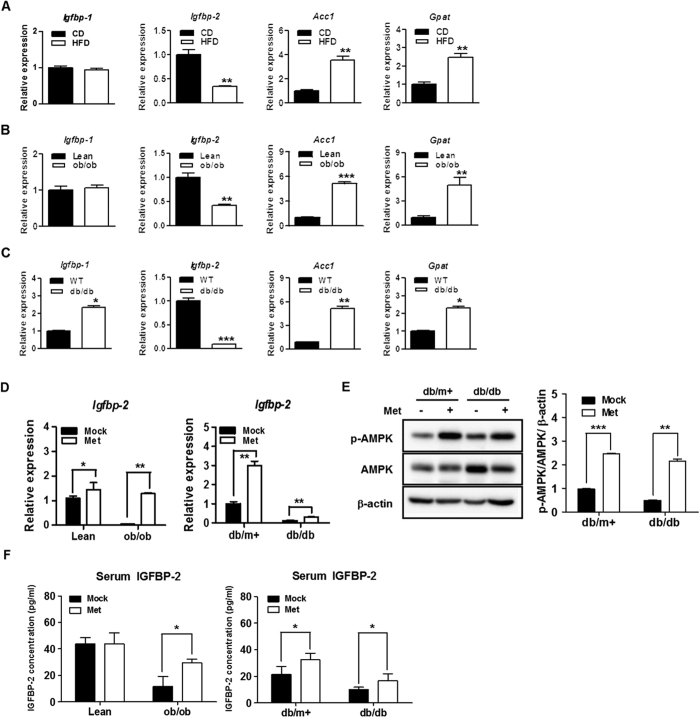
Hepatic *Igfbp-2* gene expression in HFD, *ob/ob*, and *db/db* mice and the effects of *in vivo* treatment with metformin on liver tissue IGFBP-2 expression in *ob/ob* and *db/db* mice. (**A**) Expression of hepatic *Igfbp-2* in response to obesity and diabetes states. Quantitative polymerase chain reaction (qPCR) analysis of total RNAs from liver of chow diet (CD) and high-fat diet (HFD) mice (*n* = 5). (**B**) Hepatic expression of *Igfbp-2* in liver from *ob/ob* mice (*n* = 5). (**C**) Relative *Igfbp-2* mRNA expression in liver tissue of *db/db* mice (*n* = 5). *p < 0.05, **p < 0.01, ***p < 0.001 vs. CD or Lean or WT mice. Metformin was administered to lean, *ob/ob, db/m*+, and *db/db* mice for 3 weeks (100 mg/kg/day). (**D**) Total RNA was isolated from liver tissues of the mouse and the levels of IGFBP-2 mRNA expression were quantified by quantitative real-time PCR. (**E**) Proteins were extracted from liver tissue of the *db/m*+ and *db/db* mouse administered with water or metformin. AMPK phosphorylation were detected by immunoblotting. (**F**) Blood sample was collected from lean, *ob/ob, db/m*+, and *db/db* mouse treated with water or metformin. Secretion levels of IGFBP-2 were measured by ELISA. *p < 0.05, **p < 0.01, ***p < 0.001 vs. vehicle administered control.

To determine the potential role of *Igfbp-2* in obese and diabetic states, we observed the expression level in mouse liver. *Igfbp-2* expression was significantly decreased in the livers of high-fat diet-fed (HFD), *ob/ob* and *db/db* mice (*p* < 0.05); however, *Igfbp-1* was not significantly different. Lipogenic gene expressions (*Acc*1 and *Gpat*) were increased in HFD, *ob/ob*, and *db/db* mice, which were measured as positive controls (Fig. 2A–C). Interestingly, the expression level of hepatic *Igfbp-1* as well as lipogenic genes was significantly increased in *db/db* mice (Fig. 2C). To observe the effects of metformin on IGFBP-2 level *in vivo, ob/ob* and *db/db* mice were treated by oral gavage with 100 mg/kg/day metformin for 3 weeks and observed increased IGFBP-2 level from serum and livers. Metformin administration was induced significantly an increase of *Igfbp-2* mRNA level in the livers of *ob/ob* and *db/db* mice and phosphorylation of AMPK protein was also increased in the livers of metformin-administrated lean and *db/db* mice (Fig. 2D,E). As was the case with the mRNA expression, serum levels of IGFBP-2 were also significantly increased in the metformin-fed mice, particularly in the *ob/ob* and *db/db* mouse rather than their littermates control groups (Fig. 2F). Taken together, these results suggested that *Igfbp-2* might be involved in the development obesity- and diabetes-associated metabolic dysfunction of the liver.

### Metformin-induced Igfbp-2 gene expression is mediated by AMPK

**Figure 3:**
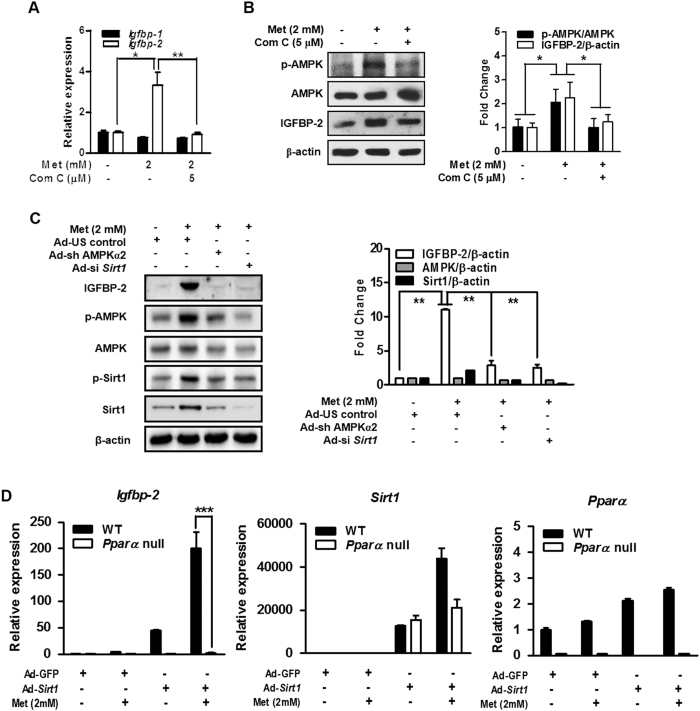
Metformin-induced *Igfbp-2* gene expression is mediated by AMPK. (**A**) *Igfbp-1* and *Igfbp-2* mRNA expressions in mouse primary hepatocytes treated with metformin and compound C (Com C) for 12 h. (**B**) Effect of metformin and compound C (Com C) on the expression of IGFBP-2 and AMPK in primary hepatocytes. The bar graph on the right shows the quantification of IGF-1 phosphorylation over total IGF-1. (**C**) Effect of metformin on IGFBP-2 protein level when *Ampk* or *Sirt1* was knocked down. AML12 cells were infected with Ad-si *Ampk*α*2* and Ad-si *Sirt1* for 36 h and then treated with metformin for 24 h. IGFBP-2, Sirt1 and AMPK phosphorylation in AML12 cell line for the indicated conditions were detected by immunoblotting. *p < 0.05, **p < 0.01 vs. untreated control and/or metformin-treated cells. (**D**) Relative hepatic *Igfbp-2, Sirt1*, and *Ppara* mRNA level in the primary hepatocytes from WT and *Ppara* null mice transfected with Ad-GFP or Ad-*Sirt1*. Mouse primary hepatocytes from WT and *Ppara* null mice were infected with Ad-GFP or Ad-*Sirt1* for 24 h and then treated with metformin for 12 h. ***p < 0.001 vs. WT mice.

To determine whether metformin-mediated AMPK activation is involved in the induction of *Igfbp-2*, we examined the effect of metformin-AMPK pathway on IGFBP-2 after treating primary hepatocytes with metformin and compound C (Com C), an AMPK inhibitor. Com C significantly repressed metformin-mediated induction of *Igfbp-2* mRNA and protein (Fig. 3A,B). Next, to confirm that metformin-mediated *Igfbp-2* expression occurs through AMPK, we adopted adenovirus-mediated gene silencing of AMPK (Ad-si *Ampkα2*) in the AML12 cell line. As shown in Fig. 3C, Ad-si *Ampkα2* significantly decreased IGFBP-2 protein level in the AML12 cells treated with metformin. In addition, Ad-si *Sirt1* treatment also decreased metformin-induced IGFBP-2 protein level (Fig. 3C). To further demonstrate effect of AMPKα1 on *Igfbp-2* gene regulation, mouse primary hepatocytes were infected with adenovirus overexpressing dominant negative form of AMPK (Ad-DN-*Ampk*). As expected, up-regulation of *Igfbp-2* mRNA and protein levels by metformin were markedly attenuated by Ad-DN-*Ampk* when compared with that of the controls (Supplementary Fig. 1). In addition, we measured *Ampkα1* and *Ampkα2* mRNA expression levels in the Ad-si *Sirt1* infected ALM12 cell line and Ad-*Sirt1* infected mouse primary hepatocytes with metformin treatment to confirm whether *Sirt1* expression affects *Ampk* expression. Increased *Ampkα1* and *Ampkα2* mRNA expression levels by metformin were significantly decreased by *Sirt1* depletion and were increased by *Sirt1* overexpression (Supplementary Fig. 2). Next, to confirm that metformin mediates *Igfbp-2* expression through PPARα, we overexpressed *Sirt1* using adenovirus with metformin treatment in *Pparα* null primary hepatocytes and measured *Igfbp-2* expression level. As expected, in *Pparα* null cells, even when *Sirt1* is overexpressed, metformin was unable to increase *Igfbp-2* mRNA expression level (Fig. 3D). This result suggests that Sirt1 may be involved in regulation of *Igfbp-2* expression essentially through PPARα. Collectively, these results indicate that AMPK plays a major role in regulating metformin-mediated stimulation of *Igfbp-2* expression in primary hepatocytes and the AML12 cell line and *Sirt1* could be also involved in the transcriptional regulation of metformin-mediated *Igfbp-2* gene expression.

### Sirt1 controls Igfbp-2 expression via PPARα-dependent pathway

**Figure 4:**
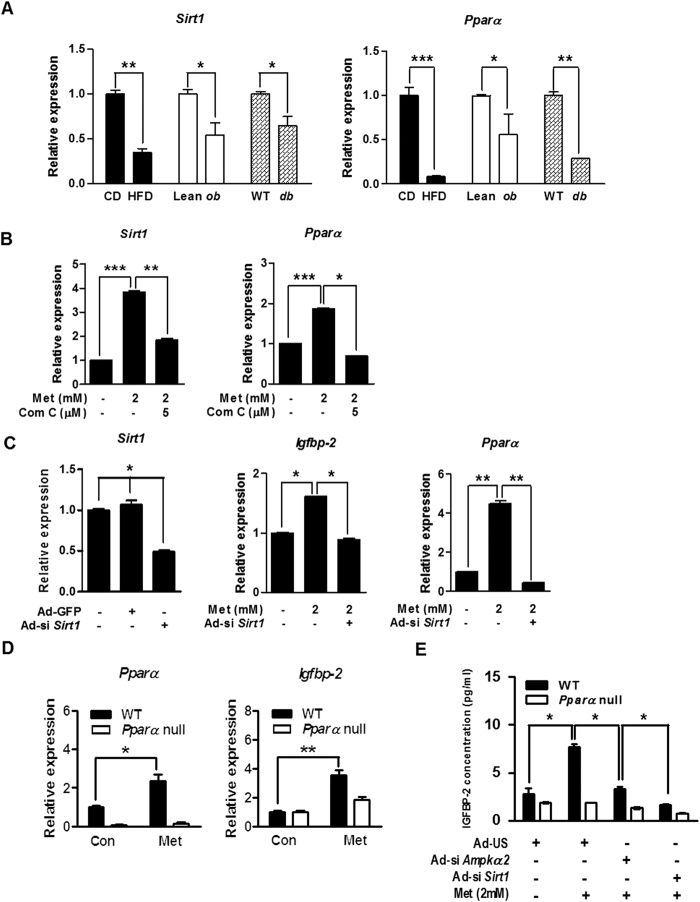
*Sirt1* controls *Igfbp-2* expression via a *Ppar*α-dependent. (**A**) Relative *Sirt1* and *Ppar*α mRNA expression in liver tissue from HFD, *ob/ob*, and *db/db* mice (*n* = 5). *p < 0.05, **p < 0.01, ***p < 0.001 vs. CD or Lean or WT mice. (**B**) Expression of hepatic *Sirt1* and *Ppar*α in mouse primary hepatocytes treated with metformin and compound C (Com C) for 12 h. (**C**) qPCR analysis of hepatic *Sirt1, Igfbp-2,* and *Ppar*α mRNA level in the primary hepatocytes transfected with Ad-si *Sirt1.* Mouse primary hepatocytes were infected with Ad-si *Sirt1* for for 36 h and then treated with metformin for 12 h. (**D**) Mouse primary hepatocytes from WT and *Ppar*α null mice were incubated in the presence or absence of metformin for 12 h *Igfbp-2* and *Ppar*α mRNA expression were measured by qPCR analysis. (**E**) Secretion level of IGFBP-2 *in vitro*. Mouse primary hepatocytes from WT and *Ppar*α null mice were infected with Ad-si *Ampk*α*2* and Ad-si *Sirt1* for 36 h and then treated with metformin for 24 h. Supernatant medium were collected and secretion levels of IGFBP-2 were measured by ELISA. *p < 0.05, **p < 0.01, ***p < 0.001 vs. untreated control or metformin-treated cells.

To further confirm the relationship between *Sirt1* and *Igfbp-2* gene expression *in vivo*, we performed qPCR analysis on liver samples obtained from obese and diabetic mice. The expression levels of *Sirt1* and *Pparα* mRNA were significantly decreased in the obese and diabetic mice compared to respective control groups (Fig. 4A). Metformin significantly elevated *Sirt1* and *Pparα* mRNA levels which were markedly diminished by Com C in primary hepatocytes (Fig. 4B). Next, we investigated whether Sirt1 is also involved in the regulation of *Igfbp-2* expression by silencing *Sirt1* (Ad-si *Sirt1*). We infected adenovirus of si *Sirt1* in primary hepatocytes. Metformin significantly elevated *Igfbp-2* and *Pparα* mRNA levels, and this stimulatory effect of metformin was markedly attenuated by Ad-si *Sirt1* (Fig. 4C). Interestingly, the increase in the mRNA levels of *Igfbp-2* and *Pparα* was observed in metformin-administered wild-type (WT) mice but not in *Pparα*-null mice (Fig. 4D). Furthermore, metformin increased IGFBP-2 secretion in primary hepatocytes isolated from WT mice, whereas *Ampk*α*2*- or *Sirt1*-silenced primary hepatocytes exhibited significantly reduced IGFBP-2 secretion compared with primary hepatocytes from *Pparα*-null mice (Fig. 4E). Taken together, these results suggest that the metformin-AMPK-Sirt1 signaling network controls *Igfbp-2* gene expression in a PPARα-dependent manner.

### Metformin regulates PPARα-mediated Igfbp-2 gene promoter activity

**Figure 5:**
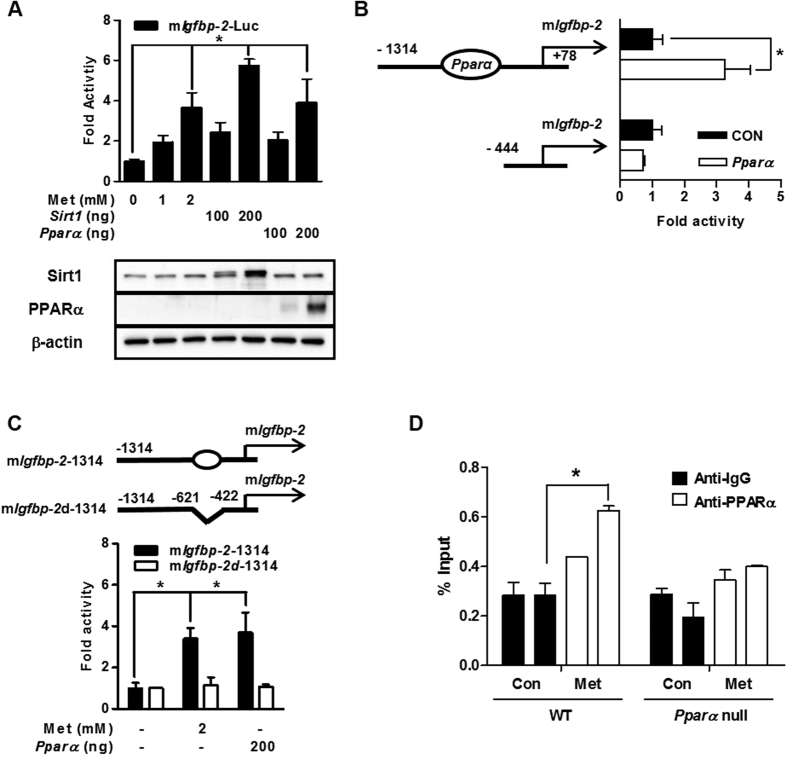
Metformin regulates PPARα-mediated *Igfbp-2* gene expression. (**A**) Effect of the *Ppar*α on mouse *Igfbp-2* promoter activity. Transient transfections were performed using observed promoter construct, *Igfbp-2* (−1314/+78) in 293T cell. Below, immunoblot analysis of *Sirt1* and *Ppar*α expression in transfected HEK293T cells, detected with anti-Sirt1 and PPARα. Equal amounts of protein were confirmed using β-actin. (**B**) Schematic drawing of *Ppar*α binding region of *Igfbp-2* promoter. Serial deletion constructs of *Igfbp-2* promoter (−698/−686 bp) were transiently transfected with *Ppar*α to 293T cell. (**C**) PPRE-dependent activation of *Igfbp-2* promoter activity. Alignment of potential PPRE in *Igfbp-2* promoter is indicated (top panel). (**D**) ChIP assay. Mouse primary hepatocytes were treated metformin for 24 h. Chromatin was precipitated using an anti-PPARα antibody from primary hepatocytes, and purified DNA samples were used to perform qPCR with primers binding to the PPRE regions on the *Igfbp-2* gene promoter. Input represents 10% of purified DNA in each sample. *p < 0.05 vs. untreated control or individual-treated cells. m*Igfbp*-2, mouse *Igfbp*-2.

To investigate whether metformin modulates the promoter activity of mouse *Igfbp-2*, we performed transient transfection reporter assay using mouse *Igfbp-2*-Luc (m*Igfbp-2*-Luc) and *Sirt1* and *Pparα* expression vectors. As expected, the transcriptional activity of *Igfbp-2* gene promoter activity was significantly increased by metformin treatment and by overexpression of *Sirt1* or *Pparα* genes (Fig. 5A). Serial deletion and/or PPRE deletion of the *Igfbp-2* promoter was performed. Promoter activity of *Igfbp-2* gene was increased by overexpression of *Ppar*α and truncations of the PPRE on the promoter resulted in a loss of PPARα responsiveness (Fig. 5B). Moreover, metformin and PPARα-mediated activation of *Igfbp-2* gene promoter activity was decreased by internal deletion of PPRE on full length of *Igfbp-2* gene promoter (Fig. 5C). ChIP assays of mouse primary hepatocytes in the presence of metformin showed that PPARα occupancy was greater on the *Igfbp-2* promoter when metformin was treated (Fig. 5D). These results strongly suggest that metformin positively regulates the *Igfbp-2* promoter through PPARα occupancy.

### Metformin controls IGF-1 signaling cascade in primary cultured hepatocytes

**Figure 6:**
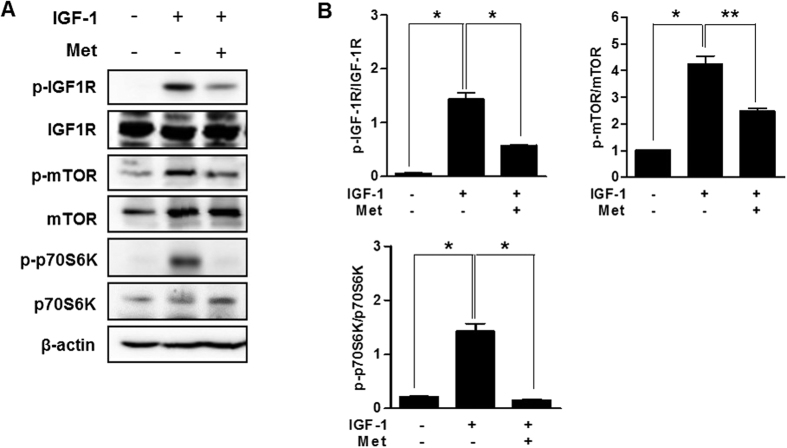
Metformin controls IGF-1 signaling cascade in primary cultured hepatocytes. (**A**) Mouse primary hepatocytes were pretreated with metformin for 12 h and then exposed to IGF-1 for 15 min at the indicated conditions. Whole cell extracts were isolated from primary hepatocytes of the indicated groups and measured by western blot analysis with various antibodies. (**B**) The left panel reveals the quantification of IGF-1 phosphorylation over total IGF-1, right panel indicates mTOR phosphorylation over total mTOR, and bottom panel indicates p70S6K phosphorylation over total p70S6K. *p < 0.05, **p < 0.01 vs. untreated control or IGF-1-treated cells.

To evaluate the potential effect of PPARα on hepatic IGF-1 bioactivity, we treated primary hepatocytes isolated from mice which were treated with IGF-1 and metformin. Metformin decreased IGF-1-induced IGF-1R and mTOR phosphorylation in primary cultured hepatocytes (Fig. 6A,B). These findings further support our data showing that metformin regulates the IGF-1 signaling system.

### Metformin improves IGF-1 bioavailability in diabetic patients

**Figure 7:**
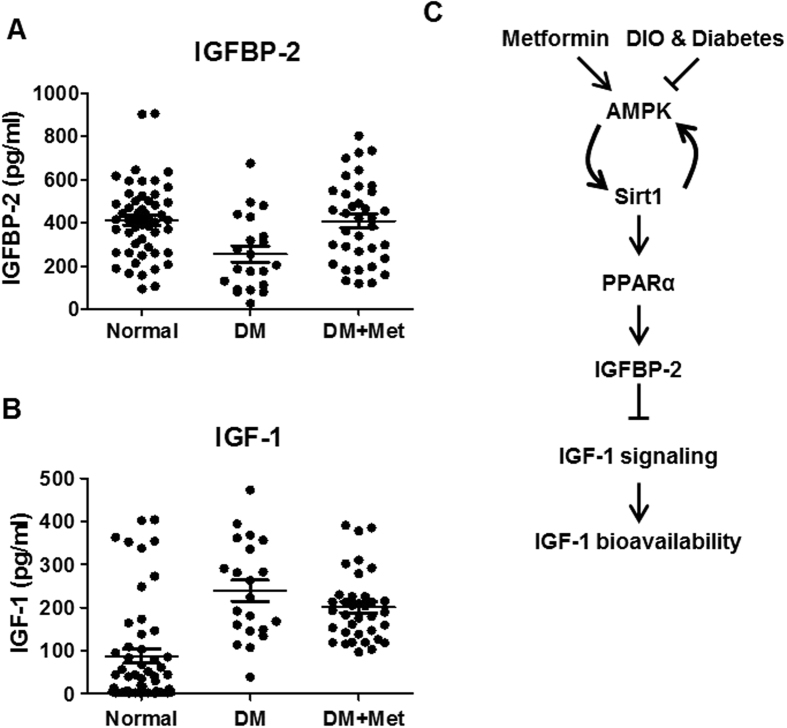
Metformin improves IGF-1 bioavailability in diabetic patients. Serum IGFBP-2 (**A**) and IGF-1 (**B**) in the control normal, diabetes (DM), and metformin-treated diabetes patients (DM + Met). (**C**) Scheme of the role of metformin in the regulation of IGFBP-2-IGF-1 signaling network. Metformin elevates *Igfbp-2* gene expression though AMPK-Sirt1-PPARα signaling pathway and then improves IGF-1 bioavailability via attenuation of IGF-1 signaling. However, the beneficial effects of metformin are counteracted in diet-induced obesity and diabetes states.

Finally, we attempted to elucidate the relationship between metformin and hormonal profile in diabetic patients whose biochemical characteristics of patients were recorded. The anthropometric measurements and biochemical characteristics of all study groups are summarized in Table 1, and the hormonal profiles of patients and control individuals are listed in Table 2. Blood sugar, HbA1c, markers of liver function (alanine transaminase and aspartate aminotransferase), lipid parameters (triglycerides, cholesterol, low-density lipoprotein and non-esterified fatty acid), and circulating C-reactive protein levels were significantly elevated in diabetic patients relative to control groups. These metabolic parameters were well ameliorated in patients group who took metformin. Indeed, serum IGFBP-2 level was markedly reduced in diabetic patients. On the contrary, circulating IGFBP-2 level of metformin treatment group was restored to similar with that observed in control group (Fig. 7A). Interestingly, even though IGF-1 level is huge increased in diabetic patients, metformin treatment did not suppress an increased IGF-1 level in diabetic patients (Fig. 7B). These findings suggest that metformin plays a pivotal role in regulating IGF-1 bioavailability without reducing total IGF-1 level in diabetic patients.

## Discussion

In this study, we demonstrate that metformin increases *Igfbp-2* expression through the AMPK-Sirt1-PPARα signaling cascade both *in vitro* and *in vivo*. Conversely, these effects of metformin were attenuated by si *Ampk*α*2* overexpression as well as silencing of *Sirt1* and *Ppar*α genes. Further analysis revealed that metformin stimulates the *Igfbp-2* promoter activity by PPARα. Previously we have reported the presence of PPARα response element on *Igfbp-2* promoter that contributes to fasting induction of IGFBP-2^27^. Based on these data, we speculated that metformin decreased IGF-1 action by upregulating *Igfbp-2* gene expression through an AMPK-Sirt1-PPARα signaling network.

Circulating IGFBP-2 levels are correlated with metabolic dysfunction including obesity, diabetes, and insulin resistance as *Igfbp-2* gene expression is lower in obese and diabetic patients^28^. IGFBP-2 is a negative regulator of IGF-1 homeostasis^7^,^8^,^29^. A previous study showed IGFBP-2 concentrations were lower in serum of mildly insulin-resistant glucose-tolerant patients^28^. Also, restoration of IGFBP-2 in insulin-resistant patients is associated with improved insulin sensitivity^30^,^31^. These clinical observations are consistent with the results of our study, where a decrease in *Igfbp-2* expression and serum levels were observed in both diet-induced obese and diabetic animal models as well as in diabetic patients compared to control groups. IGF-1 levels were also significantly higher in diabetic patients. From these findings, we propose that IGFBP-2 may modulate hepatic metabolic dysfunction by regulating IGF-1 homeostasis.

As mentioned previously, *Sirt1* is associated with metabolic diseases involving the IGF-1 signaling system in the liver^12^,^32^,^33^. However, the connection between the IGFBP-2-IGF-1 axis and the beneficial effects of *Sirt1* in the liver was not previously understood. We demonstrated that metformin significantly elevated hepatic *Sirt1* gene expression in an AMPK-dependent manner. We verified that metformin-induced *Igfbp-2* expression was mediated by the Sirt1-dependent pathway and the effect of metformin was disrupted by gene silencing of *Sirt1*. Thus, our studies strongly suggest that regulation of the metformin-AMPK pathway through Sirt1 plays an important role in ameliorating hepatic metabolic dysfunction by controlling *Igfbp-2* gene expression. However, there is a report suggesting that Sirt1 affects AMPK activation^34^. In our data, we showed that metformin-induced *Ampkα1* and *Ampkα2* mRNA expression levels were decreased by knockdown of *Sirt1* and were increased by *Sirt1* overexpression (Supplementary Fig. 2). These results indicated that AMPK expression might be also regulated by Sirt1 in a feedback regulatory mechanism.

PPARα acts a key role in regulating diverse metabolic processes and maintaining metabolic balance in response to changes in fuel availability^14^,^15^. A recent study demonstrated that AMPK activators such as metformin and panduratin A control diet-induced obesity and inflammation through the PPARα pathway in the liver^35^. Therefore, it has been suggested that AMPK activators, including resveratrol, EGCG, and other natural products, might have beneficial effects on hepatic metabolic dysfunction. We also demonstrated that the metformin-AMPK-PPARα pathway significantly activates *Igfbp-2* expression in mice and that metformin treatment is correlated with increased serum levels of IGFBP-2 in human patients.

This report suggests that metformin may be involved in IGF-1 homeostasis in response to metabolic dysfunction, and this effect was disrupted by gene silencing of AMPK expression and in *Pparα*-null mice. Our results imply that activation of AMPK and PPARα in human patients might be possible to regulate the IGFBP-2-IGF-1 axis. Meanwhile, there is possibility that another signaling pathway might exist between the IGFBP-2-IGF-1 axis and the metformin-AMPK-PPARα pathway in the liver. Moreover, interestingly, we showed *Ppar*α expression level was increased by metformin treatment in primary hepatocytes and AML12 cells. Indeed there were putative transcription factors binding sites on the *Ppar*α promoter. Even though we did not provide a mechanistic mechanism for how metformin actually induced *Ppar*α gene expression here, the signaling system underlying these effects of metformin needs to be elucidated in future studies. Also, even though IGFBP-2 secretion was completely inhibited, metformin treatment of PPAR-null cell showed slight induction of *Igfbp-2* expression (Fig. 4D). We speculated that transcription factors other than PPARα may be involved in the regulation of *Igfbp-2* gene expression. Moreover, IGFBP-2 secretion has been known to be stimulated by growth hormone^36^, but the underlying regulatory mechanism has not been addressed yet. Understanding the discrepancy between intracellular and secretory IGFBP-2 regulation could be an interesting research in the future.

In conclusion, as depicted in the novel schematic model in Fig. 7C, our current study suggests that upregulation of *Igfbp-2* by metformin activates the AMPK-Sirt1-PPARα signaling network. Furthermore, our results also indicate that metformin affects IGF-1 homeostasis by modulating IGF-1 bioavailability in the diabetic condition. These findings may aid the development of a novel pathway that could be applied to ameliorate metabolic dysfunction.

## Materials and Methods

### Reagents

Metformin (Sigma-Aldrich, St. Louis, MO, USA), compound C (Calbiochem, Billerica, MA, USA), and recombinant human IGF-1 (Life Technologies, Carlsbad, CA, USA) were purchased from the indicated companies and dissolved in the recommended solvents. Cell culture media were purchased from Gibco-BRL (Grand Island, NY, USA).

### Animal studies

All animal experiments were performed in accordance with the approved guidelines and the protocol is approved by the Institutional Animal Use and Care Committee (IAUCC), Keimyung University School of Medicine (KM-2014-33R3). Male C57BL6 mice, *ob/ob, db/db* (Jung-Ang Experimental animals, Seoul, Republic of Korea), and *Pparα* null mice (8-weeks-of-age) were used in experiments, as previously described^37^. For the diet experiment, 8-week-old male mice were fed with a LFD (10% of total calories in the form of lard fat) or HFD (60% of total calories in the form of lard fat) for 12 wks (D12450B or D12492; Research Diets, New Brunswick, NJ, USA.

### Metformin administration

The male mouse (7 weeks, *n* = 32) strains *db/m*+ (C57BLKS/J lar-m^+^/Lepr^db^), *db/db* (C57BLKS/J lar-Lepr^db^/Lepr^db^), lean and *ob/ob* (C57BL/6J Ham Slc-ob/ob) were divided into two groups: metformin (*n* = 16) and control groups (*n* = 16). In the metformin group, metformin (100 mg/kg/day) was administered by the oral for 3 weeks. In control group, vehicle (water) was administered by daily oral gavage. After the mice were fasted for 12 h at the end of the treatment period, blood samples were collected. Blood samples were used for determinations of serum IGFBP-2 levels. Real-time PCR and Western blot analysis were performed to examine IGFBP-2 mRNA and protein expression in the removed liver tissues, respectively. All animal experiments were carried out in accordance with the institutional guidelines and the all procedures and care administered were approved by the Institutional Animal Use and Care Committee (IAUCC), Keimyung University School of Medicine (KM-2012-55R and KM-2016-3R).

### Construction of plasmids and DNA

The cDNA encompassing the *Igfbp-2* open reading frame was cloned by reverse transcriptase-PCR. Primers containing *EcoR*I were used for cloning of mouse *Igfbp-2*. The PCR product was digested by EcoRI and ligated into pcDNA3 (Invitrogen, CA, USA). The reporter plasmid mouse *Igfbp-2* promoter was PCR-amplified form mouse genomic DNA (Novagen, Merck KGaA, Darmstadt, Germany) and inserted into the pGL3 Basic vector (Promega, Madison, WT, USA) using the *Sma*I and *EcoR*I restriction enzyme sites. Expression vector for *Sirt1* and *Pparα* were previously described^38^. The deletion form of mouse *Igfbp-2*-Luc was generated using PCR analysis. Plasmids were confirmed by sequencing analysis.

### Cell culture and transient transfection assays

AML-12 immortalized mouse hepatocytes were cultured in DMEM/F-12 medium (Gibco-Brl) supplemented with 10% FBS, insulin-transferrin-selenium (Gibco-BRL), dexamethasone (40 ng/mL; Sigma-Aldrich), and antibiotics in a humidified atmosphere containing 5% CO_2_ at 37 °C. HEK293T cells were cultured in Dulbecco’s modified Eagle’s medium (DMEM; Gibco-BRL, Grand Island, NY, USA) supplemented with 10% fetal bovine serum (FBS; Hyclone, Logan, UT, USA) and antibiotics in a humidified atmosphere containing 5% CO_2_ at 37 °C. Transient transfections were conducted using Lipofectamine 2000 (Invitrogen, Carlsbad, CA, USA) according to the manufacturer’s instruction.

### Isolation and culture of primary mouse hepatocytes

Mouse primary hepatocytes were isolated from the livers of 8-week-old male mice (Samtako, Osan, Republic of Korea). The protocol for isolation of hepatocytes was described previously^39^.

### Microarray expression profiling and CyberT analysis

Total RNA was prepared from each primary hepatocytes separately and equal amounts from 9 different individual samples were pooled together. Equal amounts of RNA (500 ng) from each pool were used to hybridize to triplicate microarray slides covering the entire transcriptome (Operon Whole 27K Oligo chip from Operon). The results for the triplicate chips were analyzed by GenePix operating software (GenePix pro 4.1). For statistical and biological significance, we set the Standard Deviation of Differential Expression (SD < *p*) at <0.5 and fold change at 1.5. These data were used to construct the “heat-map” using MeV v4.61 multiexperiment viewer software (http://www.tm4.org/mev).

### Recombinant adenovirus

Adenoviruses expressing US, sh *Ampk*α*2*, si *Sirt1, Sirt1,* and green fluorescence protein (GFP) have been described previously^40^,^41^.

### Measurement of mRNA

Total RNA was isolated from liver tissue and used for qPCR as previously described^37^. The expression of all transcripts using qPCR data were normalized to ribosomal L32 expression. The sequences of the primers used for gene expression analyses are reported in Supplementary Table 1.

### Immunoblotting

Proteins were isolated from liver tissues and analyzed according to the methods described previously. The membranes were probed with antibody against phospho-AMPK, AMPK, phospho-IGF-1R, phospho-Akt, Akt, phospho-Sirt1, Sirt1, phospho-p70S6K, p70S6K (Cell Signaling Technology, Danvers, MA, USA), PPARα, IGFBP-2, IGF-1R, and β-actin (Santa Cruz Biotechnology, Santa Cruz, CA, USA), and then developed using an ECL Western blot detection kit (Amersham Bioscience, Piscataway, NJ, USA).

### Human study population

This study was carried out in adherence with the guidelines of the Declaration of Helsinki and approved by the Institutional Review Boards of Keimyung University Dongsan medical center in Korea (DSMC2013-09-003-010), and informed consents were obtained from all subjects. The present study was conducted on patients with type 2 diabetes (*n* = 56) and non-diabetic control (*n* = 53) who were confirmed with insulin secretion test in the department of Internal Medicine in Dongsan medical center from October 2012 to October 2014. Among the 56 patients with type 2 diabetes, the prevalence of metformin medication was 64.3% (*n* = 36) and medication without metformin was 35.7% (*n* = 20). Exclusion criteria were: chronic alcohol drinking, hepato-biliary abnormalities, or any other acute disease.

### Human study design and assessments

A detailed questionnaire was completed for each of the 109 participating subjects. Information obtained included age, gender, height, weight, blood pressure, smoking history, history of alcohol consumption or hepatobiliary disorders, duration of diabetes, and history of hypertension or cardiovascular diseases. Additionally, fasting blood glucose (FBG), 2 hour postprandial blood glucose (2PPBG), C-peptide, insulin, insulin-like growth factors-binding protein-2 (IGFBP-2) levels were obtained. Fasting venous blood was drawn on the day of examination via a venipuncture from the subject’s antecubital vein and baseline biochemical profiles including serum total glycosylated hemoglobin (HbA1c) and glucose were analyzed using automated glycohemoglobin analyzer HLC-723G7 (Tosoh , Japan) and BIOSEN C-line, clinic (EKF Diagnostic, Germany). For serum and plasma IGFBP-2 measurement, blood samples were collected in tubes containing EDTA and IGFBP-2 concentrations were analyzed with RayBio Mouse IGFBP-2 ELISA Kit (RayBiotech, Inc., Norcross, GA, USA). This study was approved by the Institutional Review Boards of Keimyung University Dongsan medical center in Korea (DSMC2013-09-003-010), and informed consents were obtained from all subjects.

### Statistical analysis

Data calculation and statistical analyses were performed using GraphPad Prism 3–5.0 software. The statistical significance of differences between groups was determined using Student’s *t* test and multiple comparisons were analyzed using one-way ANOVA under treatment and experiment as factors. Results are presented as mean ± standard deviation of at least three separate experiments. All *P* values less than 0.05 were considered significant.

## Additional Information

**How to cite this article**: Kang, H. S. *et al*. Metformin stimulates IGFBP-2 gene expression through PPARalpha in diabetic states. *Sci. Rep.* **6**, 23665; doi: 10.1038/srep23665 (2016).

## Supplementary Material

Supplementary Information

## Figures and Tables

**Table 1:** Clinical characteristics of the participants.

Characteristic	Nor	DM	DM + Met
*N*(M/F)	53 (17/36)	20 (9/11)	36 (21/15)
Age (years)	58.33 ± 3.83	54.31 ± 3.36	63.53.31 ± 2.95
Height (cm)	158.81 ± 1.95	163.74 ± 1.77	159.92 ± 2.12
Weight (g)	62.02 ± 2.26	67.62 ± 3.38	65.13 ± 3.15
BMI (kg/m^2^) (male)	24.03 ± 3.03	26.16 ± 0.89	25.6 ± 3.13
BMI (kg/m^2^) (female)	24.96 ± 2.84	23.24 ± 1.56	24.88 ± 4.68

**Table 2:** Biochemical characteristics of the participants with metformin-challenged type2 diabetes mellitus.

Characteristic	Nor	DM		DM + Met	
FBS (mg/dl)	94.58 ± 5.16	132.92 ± 13.51	**	117.53 ± 7.88	**
HbA1c (mmol/l)	6.18 ± 0.13	8.16 ± 0.62	*	6.85 ± 0.15	*
Insulin (pmol/l)	32.56 ± 4.97	24.08 ± 4.38	**	24.73 ± 2.66	
C-peptide	10.4 ± 0.87	8.03 ± 1.1	*	7.9 ± 0.97	
ALT (IU/l)	26.17 ± 5.27	28.69 ± 4.44	*	19.8 ± 2.51	**
AST (IU/l)	30.83 ± 2.8	25.23 ± 3.38	*	20.4 ± 1.17	*
TG (mg/dL)	147.36 ± 38.4	152.22 ± 17.2	*	118.69 ± 14.64	**
Chol (mg/dl)	165.75 ± 11.06	193.38 ± 12.43	**	164.53 ± 11.22	**
HDL (mg/dl)	48.43 ± 3.01	44.73 ± 3.88	*	43.87 ± 1.74	
LDL (mg/dl)	96.35 ± 9.88	113.28 ± 10.87	*	101.87 ± 9.2	**
CRP (mg/dl)	3.03 ± 1.31	9.59 ± 6.51	***	1.39 ± 0.51	***
NEFA (μEg/l)	762.08 ± 125.83	562.31 ± 80.32	**	522.13 ± 78.28	*

Nor-Normal; DM-Diabetes mellitus; Met-Metformin.

FBS-fasting blood sugar; ALT-Alanine aminotransferase; AST-Aspartate aminotransferase.

TG-Triglyceride; Chol-Cholesterol; HDL-High-density lipoprotein; LDL-Low Density lipoprotein; CRP-C-reactive protein; NEFA-Non-esterified fatty acids.

Data presented as Mean ± SEM, n = 12–15, *p < 0.05, **p < 0.01, ***p < 0.001 vs. Nor or DM.
