# Supplementary material for: Metformin stimulates IGFBP-2 gene expression through PPARalpha in diabetic states
Source: Sci Rep. 2016 Mar 24;6:23665. doi: 10.1038/srep23665 (PMC4806307; doi:10.1038/srep23665)
Supplement: Supplementary Information [file srep23665-s1.pdf]

Supplementary Online Material for

Metformin stimulates IGFBP-2 gene expression through PPARalpha in diabetic states

Hye Suk Kang, Ho-Chan Cho, Jae-Ho Lee, Goo Taeg Oh, Seung-Hoi Koo, Byung-Hyun Park, In-Kyu Lee, Hueng-Sik Choi, Dae-Kyu Song, Seung-Soon Im

Correspondence should be addressed to Seung-Soon Im (ssim73@kmu.ac.kr)

Supplementary material and method

Recombinant adenovirus. Adenoviruses overexpressing dominant negative (DN)-*Ampk*α*1* has been described previously[1].

Supplementary references

1. Woods, A., *et al.* Characterization of the role of AMP-activated protein kinase in the regulation of glucose-activated gene expression using constitutively active and dominant negative forms of the kinase. *Molecular and cellular biology* **20**, 6704-6711 (2000).

Supplementary Figure 1. Metformin-induced *Igfbp-2* gene expression is mediated by AMPKα1. (a) Expression of hepatic *Igfbp-2* in mouse primary hepatocytes. Mouse primary hepatocytes were infected with Ad-DN-*Ampkα1* for 36 h and then treated with metformin for 12 h. **(b)** IGFBP2 protein level and AMPK phosphorylation in mouse primary hepatocytes for the indicated conditions. * $p < 0.05$ vs. untreated control and/or metformin-treated cells.

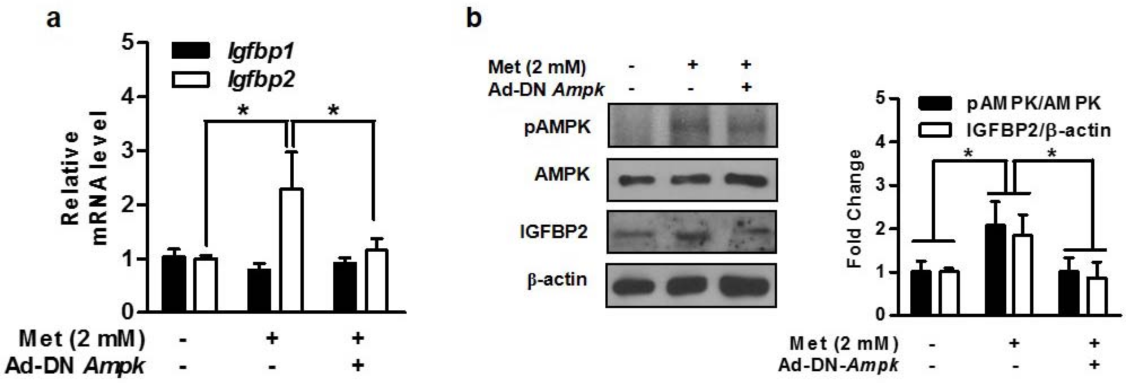

Supplementary Figure 2. AMPKα expression is affected by Sirt1 expression level. (a) Effect of *Sirt1* depletion on expression of *AMPKα1 and AMPKα2*. AML12 cells were infected with Ad-US or Ad-si *Sirt1* for 36 h and then treated with metformin for 12 h. **(b)** Effect of *Sirt1* overexpression on *AMPKα1 and AMPKα2* expression. Mouse primary hepatocytes were infected with Ad-GFP or Ad- *Sirt1* for 24 h and then treated with metformin for 12 h. *$p < 0.05$, ** $p < 0.01$, *** $p < 0.001$ vs. untreated control.

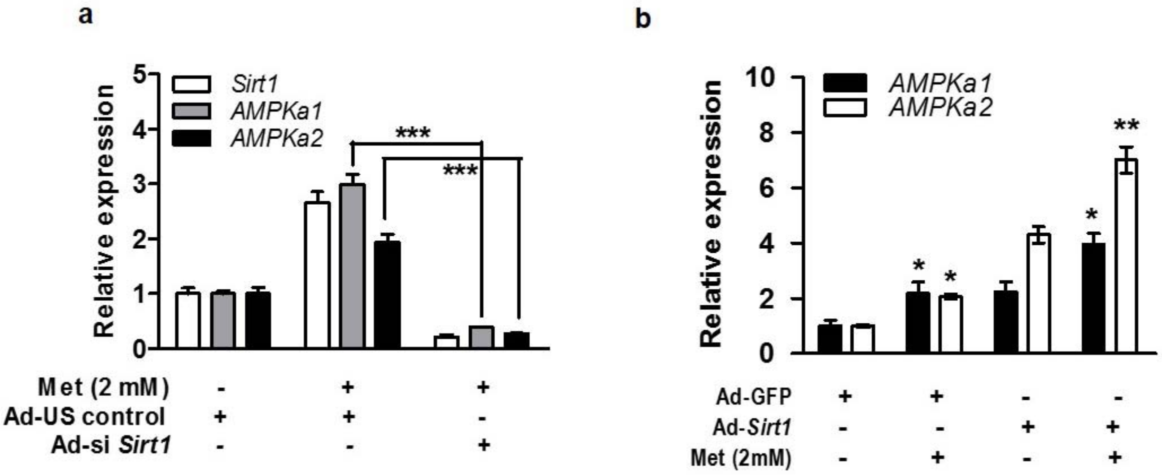

Supplementary Table 1. List of primers employed for gene expression analysis by real-time PCR.

	Forward primer (5'-3')	Reverse primer (5'-3')
Igfbp-1	ATCAGCCCATCCTGTGGAAC	TGCAGCTAATCTCTCTAGCACTT
Igfbp-2	ACCCCTTGCCAGCAGGAGTTGGA	TCCCTGGATGGGCTTCCCGGT
Acc1	TGACAGACTGATCGCAGAGAAAG	TGGAGAGCCCCACACACA
Gpat	CAACACCATCCCCGACATC	GTGACCTTCGATTATGCGATCA
Ppara	AGAGCCCCATCTGTCCTCTC	ACTGGTAGTCTGCAAAACCAAA
Sirt1	GCAGATTAGTAGGCGGCTTG	TCTCCATCAGTCCCAAATCC
AMPKa1	GTCAAAGCCGACCCAATGATA	CGTACACGCAAATAATAGGGGTT
AMPKa2	TCCTGAAGACCCCTCCTACG	GAGTGGTTCTCAGCTGTGCT
L32	ACATTTGCCCTGAATGTGGT	ATCCTCTTGCCCTGATCCTT
